# Feigning Acute Intermittent Porphyria

**DOI:** 10.1155/2014/152821

**Published:** 2014-11-25

**Authors:** Rania Elkhatib, Modupe Idowu, Gregory S. Brown, Yasmeen M. Jaber, Matthew B. Reid, Cheryl Person

**Affiliations:** ^1^Department of Psychiatry and Behavioral Sciences, University of Texas Health Science Center at Houston, 5656 Kelley Street, Houston, TX 77026, USA; ^2^Department of Internal Medicine, Division of Hematology, University of Texas Health Science Center at Houston, 5656 Kelley Street, Houston, TX 77026, USA; ^3^University of Texas Health Science Center at Houston, 1941 East Road, Houston, TX 77054, USA

## Abstract

Acute intermittent porphyria (AIP) is an autosomal dominant genetic defect in heme synthesis. Patients with this illness can have episodic life-threatening attacks characterized by abdominal pain, neurological deficits, and psychiatric symptoms. Feigning this illness has not been reported in the English language literature to date. Here, we report on a patient who presented to the hospital with an acute attack of porphyria requesting opiates. Diligent assessment of extensive prior treatment records revealed thirteen negative tests for AIP.

## 1. Introduction

The acute porphyrias are distinct genetic disorders of heme biosynthesis characterized by acute life-threatening attacks associated with nonspecific neurovisceral symptoms. Defects in each of the four different enzymes account for the characteristics of the four types of acute porphyrias. AIP is the most common of the acute porphyrias and is due to a deficiency of the heme biosynthetic enzyme porphobilinogen deaminase (PBGD) [1]. The other three acute porphyrias are hereditary coproporphyria, variegate porphyria, and aminolevulinic (ALA) dehydratase deficiency.

The four types of acute porphyria present with similar acute neurovisceral presentations and may be indistinguishable during an acute attack. The most common symptoms are gastrointestinal (acute abdominal pain, nausea, vomiting, and constipation) and neurologic (peripheral neuropathy, autonomic instability and neuropsychiatric symptoms) abnormalities.

The diagnosis of acute porphyria is very challenging since the symptoms seen in this disorder are nonspecific and are commonly seen in other medical conditions. Delaying diagnosis and treatment of acute porphyric attacks can be fatal or can result in permanent neurologic damage; hence, a high index of suspicion is imperative [2]. Initial rapid testing for urine PBG should be considered for patients presenting with symptoms of acute porphyria.

Here we identify a patient with multiple emergency department visits who, despite stating that he had been diagnosed with AIP, was without any medical testing to support the diagnosis.

## 2. Case Presentation

Mr. S, a 48-year-old divorced Hispanic male with a self-reported history of chronic pain secondary to AIP and bipolar disorder, presented to the emergency department complaining of severe abdominal pain and stated that he would return home and “shoot myself if the pain does not go away.” The patient was discharged a few hours prior to admission from a sister hospital with complaints of a “porphyria attack” and requesting opiate analgesics. He specifically asked for Dilaudid stating nothing else works for his porphyria pain. On evaluation at the outside hospital, his pulse was 76 beats per minute (bpm), blood pressure was 131/79 mmHg, and temperature was 97.9 degrees Fahrenheit. Abdominal exam was benign: soft with bowel sounds present, diffusely tender but without rebounding or guarding. He was then evaluated by a multidisciplinary psychiatry service at that time and only had an irritable mood: no suicidal ideation, no psychosis, and no manic behaviors, and he was quite future-oriented and looking forward to receiving assistance for housing and free pain medications. His discharge diagnosis was adjustment reaction with anxiety and included nonopiate medication and follow-up hematology appointment. He came directly to our hospital after discharge.

In addition to suicidal ideations, Mr. S endorsed nausea, vomiting, abdominal pain, and homicidal ideations towards his landlord. He reported that he recently moved to Texas but gave inconsistent reasons as to why he moved. Initially he stated he moved to work with a porphyria specialist here but was unable to identify the physician or the hospital where this person worked. Then he stated he moved here from New York for warmer weather but later stated he moved here from Virginia. He endorsed a depressed mood, decreased sleep, anhedonia, and poor energy. He reported an unstable housing situation and was quite angry that his landlord may have “cheated” him out of a deposit on rent. Due to this he wanted to kill the landlord. He also explained that he was only suicidal due to the pain from his porphyria. Physical exam at our hospital revealed pulse 79 bpm, blood pressure 104/72 mmHg, and temperature 97.8 degrees Fahrenheit. The abdominal exam revealed an inconsistently tender, nondistended abdomen with normal bowel sounds and absent rigidity, rebound, and guarding. Motor strength was 5/5 in all extremities. Sodium was 141 millimoles/Liter (mmol/L). He did not appear to be responding to internal stimuli throughout the evaluation. He endorsed an anxious mood but otherwise calmly described his intentions to end his life if we did not adequately control his pain. He also stated to the nursing staff that he would go and get a gun and “kill you all” if he were discharged to home. He later stated that he was only frustrated we would not give him the medicine he needed to treat his porphyria.

We obtained medical records from the hospital where a significant portion of his AIP treatment occurred. Our records begin with a diagnosis of AIP. Unfortunately, we were unable to confirm where or how his initial diagnosis was made as those records were not available. We did receive laboratory data beginning in 2004 (without an accompanying narrative) which showed 4 separate tests for AIP (see Table 1). Records we did obtain indicated that his next reported AIP flare was in September 2005, which was described as daily postprandial vomiting and twenty-pound weight loss. Although his qualitative urine porphobilinogen (PBG) was negative he was started on hemin 350 milligrams (mg) intravenously every two weeks. He also received morphine 15 mg immediate release every 6 hours as needed for pain and 100 micrograms/hour (mcg/h) of fentanyl transdermal patch.

In March 2006, he received two more hemin infusions but was poorly compliant with follow-up appointments. He presented subsequently for abdominal pain, where he was treated with intravenous hydromorphone despite two negative qualitative PBGs and negative 24-hour quantitative porphyrins. He had two more negative qualitative PBG tests in 2006 and one more 24-hour quantitative porphyrin test. In 2009 he was evaluated for abdominal pain in the emergency department (ED) and had a normal porphyrin profile at that time. In total, his urine has been tested on 11 different dates likely secondary to nine different AIP episodes to diagnose acute intermittent porphyria and each test has been negative. No genetic testing was ever received.

Mr. S was ultimately discharged from our hospital with a follow-up hematology appointment and a one-day supply of opiate analgesics. After discharge, he failed to keep any of the four outpatient hematology clinic appointments. One month later, he presented to the emergency department with a two-day history of acute abdominal pain without constipation (his last bowel movement was that morning). His vital signs were all within normal limits and his sodium was 142 mmol/L. He was discharged with three more outpatient hematology appointments which he did not attend. Since he failed to show up for any of his seven outpatient hematology appointments, we were unable to obtain any genetic testing on this patient.

## 3. Discussion

Patients with AIP are often misdiagnosed with other causes of acute abdomen and most seek treatment multiple times before the correct diagnosis is confirmed [3]. Abdominal pain secondary to neurotoxicity is among the classic signs of AIP and presents in over ninety percent of patients during an AIP attack. This excruciating pain is often associated with nausea, vomiting, hyponatremia, tachycardia, and hypertension [4]. In this case, all laboratory data available was negative and there were no additional findings beyond complaints of abdominal pain and a “porphyria attack.” While feigning symptoms for secondary gain is common for some illnesses such as chronic pain [5, 6], this presentation appears unique.

More than one hundred PBGD mutations have been described in AIP. Penetrance is incomplete and only a minority, less than 10%, of individuals with each genetic defect may have phenotypic expression of the disease. The clinical sequelae of acute porphyrias are often unmasked in the presence of endogenous or exogenous factors that promote heme biosynthesis and lead to further accumulation of porphyrins and their precursors. Acute AIP attacks are precipitated by direct or indirect increases in heme synthesis; the most common causes are drugs and hormones, as they are inducers of cytochrome P450 enzymes. Most heme synthesized in the liver is utilized for cytochrome P450 enzymes, which turn over more rapidly than other cellular hemoproteins, increasing the demand for heme synthesis [7].

Clinicians should consider this diagnosis in all adults with unexplained symptoms seen in acute porphyrias especially if the patient presents with severe abdominal pain with or without constipation, muscle weakness, hyponatremia, hypertension, tachycardia, and dark or reddish urine.

Urinary PBG level is markedly increased (20 to 200 mg/L) in patients with acute attacks of AIP, hereditary coproporphyria, and variegate porphyria. Initial rapid testing for increased porphobilinogen (PBG) in single-void urine may help establish the diagnosis promptly. An expert panel on acute porphyrias recommends the use of Trace PBG Kit (Thermo Trace/DMA, Arlington, Texas) [2]. Samples for quantitative measurement of porphyrins should be collected during an acute attack and should not be exposed to sunlight during collection, transportation, or processing. If porphobilinogen is elevated, treatment should be started immediately. To establish the type of acute porphyria, save the same urine sample for measurement of ALA, porphobilinogen, and porphyrin levels, and measure plasma porphyrin levels, fecal porphyrin levels, and erythrocyte porphobilinogen deaminase levels [1, 2].

Urinary ALA and PBG levels are variable between attacks and at times normal. PBGD activity is about 50% of normal in red blood cells (RBC) of patients with AIP confirming an individual carries the mutation and may be at risk for clinical sequelae. PBGD has two isoforms, erythroid and nonerythroid. It is estimated that about 10% of AIP patients have a normal RBC PBGD level but have diminished PBGD activity in the liver due to a mutation in the nonerythroid isoform. In unclear situations, PBGD activity and genetic testing can also be performed [1, 2].

Hereditary coproporphyria is much less frequent than AIP and is due to a deficiency of coproporphyrinogen oxidase activity. During acute attacks excessive amounts of coproporphyrins accumulate in stool, along with ALA and PBG in the urine. Variegate porphyria is due to a deficiency of protoporphyrinogen oxidase which leads to accumulation of protoporphyrins and coproporphyrins in the stool, as well as ALA and PBG in the urine. ALA dehydratase deficiency is the least common porphyria and it is the only one that has autosomal recessive mode of inheritance. ALA dehydratase deficiency leads to accumulation of large amounts of ALA in the urine without much PBG [1].

If porphobilinogen is elevated, treatment should be started immediately with hemin 3 to 4 mg intravenously daily for at least 4 days. Intravenous glucose alone (10%, at least 300 g daily) may resolve mild attacks or may be given while waiting for hemin to be available for severe attack [2]. Intravenous hemin addresses the underlying pathophysiology of AIP, by repressing hepatic delta-aminolevulinic acid (ALA) synthase activity, thus decreasing the overproduction of ALA and porphobilinogen [8].

During acute attacks, correction of dehydration and electrolyte imbalances as well as monitoring of vital capacity and expiratory flow rate is quite essential. Intravenous narcotics will be necessary to control acute pain. Supportive measures and avoidance of precipitating factors including offending drugs are of utmost importance [2].

To our knowledge, this represents the first English language report of a patient feigning symptoms of an AIP attack for secondary gain (opiate medications). We found one other study with a similar presentation, in Polish [9]. This case represents an uncommon presentation of malingering in the emergency department setting. While a misdiagnosis of AIP may be more routine in a hematology practice, this is the first case described in the psychiatric literature. In the absence of vital sign abnormality, hyponatremia, and normal neurological exam we would recommend confirmation of AIP via PBG testing prior to initiation of hemin therapy.

## Figures and Tables

**Table 1:** 

Date	Ordering specialist/chief complaint	PBG-Qual	Porphyrins 24 h urine quant	Uroporphyrin octa 24 h urine (3–25 *μ*g/24 h)	Heptacarboxyl porphyrin 24 h urine (0–7 *μ*g/24 h)	Hexacarboxyl porphyrin 24 h urine (0–6 *μ*g/24 h)	Pentacarboxyl porphyrin 24 h urine (0–7 *μ*g/24 h)	Coproporphyrin 24 h urine (25–150 *μ*g/24 h)	Porphobilinogen 24 h urine (0–0.5 mg/24 h)
3/31/2004^¶^	Heme/onc				Result below reportable range for this analyte	Result below reportable range for this analyte			

5/4/2004^¶^	Heme/onc					Result below reportable range for this analyte	Result below reportable range for this analyte		

7/15/2004	Heme/onc	Negative							

7/16/2004	Heme/onc	Negative							

9/6/2005	Heme/onc AIP attack	Negative							

5/5/2006	Heme/onc	Negative							

5/7/2006	Heme/onc	Negative	Normal^*^	4	<1	<1	<1	26	0.1

6/3/2006	Heme/onc abdominal pain, nausea, vomiting	Negative							

11/14/2006	EM unknown+	Negative							

12/30/2006	Heme/onc unknown+	Negative	Normal^*^	24	3	<1	3	128	0.3

3/27/2009^#^	Heme/onc		Normal	10	3	<1	2	75	0.1

PBG-Qual: qualitative urine porphobilinogen; heme/onc: hematology/oncology specialist; EM: emergency medicine specialist; ^*^performed at university hospital laboratory; unknown+: unknown chief complaint but ordered in the emergency department; ^#^test performed at Mayo Medical Laboratories; ^¶^performed by Nichols Institute, Quest Diagnostics, San Juan Capistrano, CA.
